# Digital Patient Reported Outcome Measures Platform for Post–COVID-19 Condition and Other Long-Term Conditions: User-Centered Development and Technical Description

**DOI:** 10.2196/48632

**Published:** 2023-10-20

**Authors:** Manoj Sivan, Román Rocha Lawrence, Paul O'Brien

**Affiliations:** 1 Leeds Institute of Rheumatic and Musculoskeletal Medicine University of Leeds Leeds United Kingdom; 2 ELAROS 24/7 Ltd Sheffield United Kingdom

**Keywords:** post–COVID-19 condition, post–COVID-19 syndrome, post-acute COVID-19 syndrome, C19-YRS, Yorkshire Rehabilitation Scale, ELAROS, chronic conditions, mobile app, digital technology, smartphone, chronic, respiratory, COVID-19, Sars-CoV-2, coronavirus, platform, patient-reported, outcome measure, outcome measures, health record, health records, report, reports, data sharing, information sharing, mobile phone

## Abstract

**Background:**

Post–COVID-19 condition (PCC), colloquially known as long COVID, is a multisystem condition characterized by persistent symptoms beyond 4 weeks after the SARS-CoV-2 infection. More than 60 million people with PCC worldwide need prompt assessment, diagnosis, and monitoring, with many requiring specialist help from a multidisciplinary team of health care professionals (HCPs). Consequently, a scalable digital system is required for both people with PCC and HCPs to capture the breadth of symptoms and their impact on health, using patient-reported outcome measures (PROMs) and patient-reported experience measures (PREMs).

**Objective:**

We aim to develop and implement a novel PCC digital PROM (DPROM) platform for (1) securely collecting PROM and PREM data from people with PCC, (2) enabling users to monitor symptoms longitudinally and assess response to treatment, (3) generating reports for the electronic health records (EHRs), (4) providing summary reports on PCC services based on national requirements, and (5) facilitating the sharing of relevant data with authorized research teams to accelerate our understanding of this new condition and evaluate new strategies to manage PCC.

**Methods:**

We (1) undertook requirement analysis with people with PCC, HCPs, and researchers to identify the needs of the DPROM platform and determine its required functionalities; (2) designed and developed a clinically useful web portal for staff and a mobile app for patients, with a web-based alternative app to improve patient and staff choice, limit the risk of digital exclusion, and account for variability across services; (3) determined the PROMs and PREMs that PCC services would prefer to use on the platform; and (4) designed the summary report function that can be generated for each user for the EHR and for reporting to national health authorities.

**Results:**

A DPROM platform to record PCC symptom profile, condition severity, functional disability, and quality of life, based on the C19-YRS (Yorkshire Rehabilitation Scale) and other PROMs and PREMs, was developed. Individual-level medical information and details on the COVID-19 illness can be captured systematically. The platform generates easy-to-understand scores, radar plots and line graphs for people with PCC to self-monitor their condition and for HCPs to assess the natural course of the condition and the response to interventions. Clinics can configure a suite of PROMs and PREMs based on their local and national service and commissioning requirements and support research studies which require large-scale data collection on PROMs. The DPROM platform enables automatic aggregate data analysis for services to undertake service evaluation and cost-effectiveness analysis. The DPROM platform generated summary report can be uploaded to the EHRs of people with PCC.

**Conclusions:**

A multifunctional DPROM platform to assess, grade, and monitor PCC has been developed. Future research will analyze the system’s usability in specialist PCC clinical services and other long-term conditions.

## Introduction

Post–COVID-19 condition (PCC), colloquially known as long COVID, refers to persistent symptoms 4 weeks after contracting COVID-19 illness [1]. The term PCC embraces the National Institute for Health and Care Excellence scientific terms [1] “ongoing symptomatic COVID-19” for symptoms at 4-12 weeks and “post-COVID syndrome” for symptoms >12 weeks, as well as the World Health Organization (WHO) [2] term “post-COVID condition” for symptoms >12 weeks. There are more than 2 million people with PCC in the UK alone and more than 60 million cases worldwide at the time of writing [3,4]. It is a multisystem condition with more than 200 symptoms reported across 10 organ systems, with the most common symptoms being breathlessness, fatigue, palpitations, dizziness, pain, brain fog (cognitive problems), anxiety, depression, posttraumatic stress, skin rash, and allergic reactions [5]. PCC in some individuals can be a remitting and relapsing condition with a protracted course causing significant long-term distress and disability [6].

Patient-reported outcome measures (PROMs) are questionnaire tools to ascertain patients’ views of their symptoms, their functional status, and their health-related quality of life [7]. PROM use in the routine clinical management of medical conditions has been shown to facilitate communication, engage patients in their care, monitor condition progression, tailor care to individual patients’ needs, and show value for money for those investing in the services [8,9]. An ideal PROM should include clinically important concepts that define the condition in the target population, assess the impact on daily life, and reflect the lived experience of those with the condition. Given the large scale, relative novelty, and multifariousness of PCC, there is a need for developing and using condition-specific PROMs to assess functioning, disability, and health [10].

A multidisciplinary team of rehabilitation professionals working with patients recovering from COVID-19 during the first wave of the pandemic developed an outcome measure called the C19-YRS (Yorkshire Rehabilitation Scale), the original version of C19-YRS [11-13]. The content validity, construct validity, and reliability of the scale has been supported by studies both in the United Kingdom and other countries [14-16] The scale reports on symptoms, symptom severity, functional disability, and overall health state in PCC, spanning all aspects of the 2001 WHO International Classification of Functioning, Disability, and Health framework [17]. A Rasch-modified version of the scale has also been developed [18]. The use of the scale has also been recommended in the National Health Service (NHS) England clinical guidance for PCC services and National Institute for Health and Care Excellence rapid guidelines [19,20]. The scale has been translated into numerous languages and is currently used in many PCC studies worldwide.

This study aims to develop and implement a novel DPROM platform for the secure collection of individual-level data that covers all aspects of the condition (PCC) and enabling people with PCC and HCPs to monitor the condition and assess response to treatments. The platform needs to enable communication between people with PCC and HCPs, should have the ability to link to electronic health records (EHRs) and must provide services with summary data in keeping with national reporting requirements.

## Methods

### Ethical Approval

The study was approved by University of Leeds School of Medicine Research Ethics (MREC 20-041) and Yorkshire & The Humber - Bradford Leeds Research Ethics Committee (21/YH/0276). All procedures followed were in accordance with the ethical standards of the responsible committees and the Helsinki Declaration 2000.

### Requirement Analysis

The digital health company, ELAROS 24/7 Ltd (ELAROS) [21] in November 2020 collaborated with a multidisciplinary team of clinicians (8 members), researchers (2 members), and people with PCC (2 members) who developed the C19-YRS to initiate the development of a digital version and rollout of the scale nationally to meet the needs of the people with PCC in the country. The paper format of the scale is free to use by anyone and was used as a basis for developing a digital version of C19-YRS for the digital platform. The company and the University of Leeds entered an agreement to license the C19-YRS scale and incorporate the scale into their planned DPROM platform which would be offered on a not-for-profit basis to public health organizations.

The concept for DPROM was developed based on understanding user needs and requirements based on interviews and discussions with the C19-YRS team and people with PCC in Leeds and clinicians providing PCC care in Airedale NHS Foundation Trust, Northern Care Alliance NHS Foundations Trust (formerly Salford Royal and Pennine Acute Hospitals NHS Trusts) and Liverpool University Hospitals Foundation NHS Trust. Additional information on needs was gathered by reviewing the emerging scientific literature by searching Google Scholar and PubMed using the keywords *COVID-19*, *symptoms*, *mobile app*, *digital platform*, *PROMS*, *e-health*, *self-monitoring*, and *self-management*.

### Digital Platform Technological Resources

Prior to the pandemic, ELAROS had developed a CE-marked Digital Bladder Diary (DBD) PROM platform [22] for the remote assessment and diagnosis of 64 unique combinations of lower urinary tract symptoms with clinical specialists at Sheffield Teaching Hospitals NHS Trust and their hosted organization, Sheffield National Institute for Health Research Devices for Dignity Co-Operative, which enabled the company to quickly pivot an existing urology-focused system into the DPROM platform by building new features and refining the system around the needs of people with PCC and PCC clinics to help clinics rapidly respond to the challenges of the various pandemic lockdowns and deliver services to people with PCC remotely.

The app-based DPROM platform is written using web technologies via the Apache Cordova (Adobe, Apache Software Foundation) framework for Google Android (minimum operating system version 7 Nougat), Apple iOS (minimum operating system iOS version 9), and through a web-based portal via a Chromium-based browser. The intention is that patients use their mobile devices, but if they cannot do so, or prefer to use a desktop or laptop, they may access a web-based version of the mobile app instead. The web-based version can also be used by HCPs to complete the PROMs on patients’ behalf during a tele-assessment if preferred by the patients, providing additional patient choice and support. Paper versions of the C19-YRS can still be completed by patients and later uploaded to the digital platform for collation with data collected via the mobile or web app.

The web portal app is cloud-based and can be accessed by several supported Chromium-based browsers, such as Chrome, Firefox, Edge, and Safari. The web app is written in a combination of SASS, HTML, JavaScript, and PHP, based around a LAMP (Linux, Apache, MySQL, PHP/Perl/Python) technology stack. The mobile device and browser communicate using a physical app server via an app program interface, which communicates with a database server also managed within the same infrastructure. All users access a single app server and database with restricted access to their treating clinic set up by ELAROS.

People with PCC are registered on the ELAROS platform using minimum patient identifiable data: name, date of birth, gender, and health ID number such as the English NHS number or Scottish Community Health Index number) by a member of staff following referral to a PCC clinic to generate unique patient login details (username and pin). These details are shared with the individual with PCC and used to log into the C19-YRS app on the web or on mobile, with or without support from their carer, guardian, or HCP. Users then complete PROMs through the app at time points defined by their clinical team, with support from automatic reminders which can be configured by the clinic. The system processes their data and stores it in a database alongside the patient’s details for staff to access and identify individual records.

**Figure 1:**
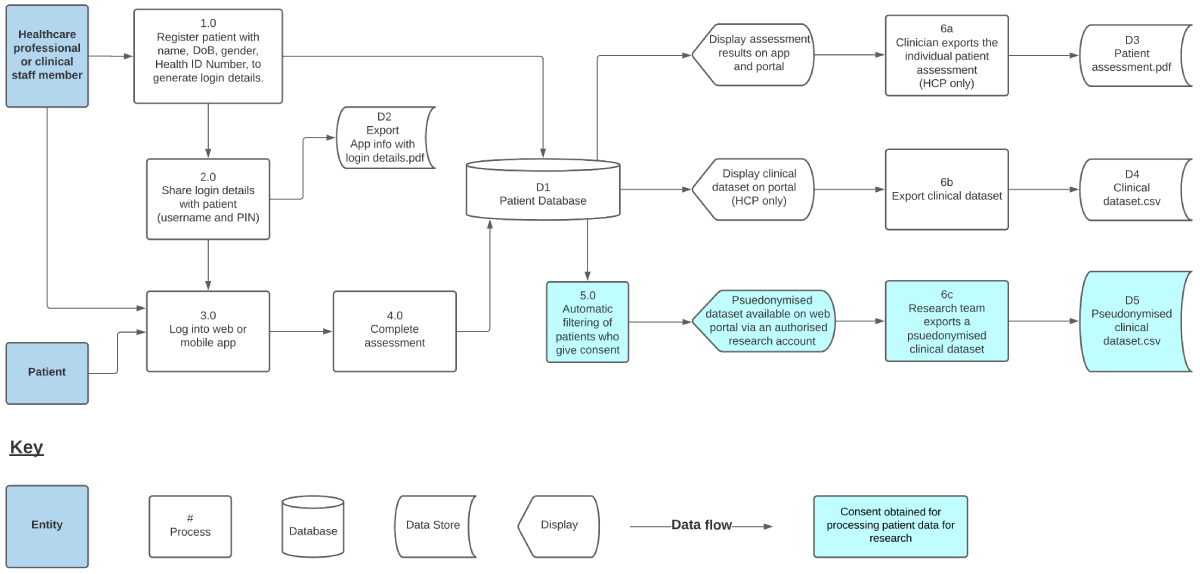
Data flow in the ELAROS digital patient-reported outcome measures platform. DoB: date of birth; HCP: health care professional; PIN: personal identification number.

The patient can access recorded data via the app to see assessment history and trends. The clinical teams can use the web portal to see the individual or overall patient reports and manage the clinic. This information can be exported into a clinically useful summary PDF report or as a comma-separated values data file which can be uploaded to the patient’s EHR, depending on the file formats the EHR accepts. A research version of the web portal is available to view pseudonymized data from patients who have consented to sharing their data with authorized researchers, enabling local or externally approved research teams to access clinical data for research at an individual, local, and or national level. This data flow is illustrated in Figure 1.

### PROMs Used by PCC Services

Few dedicated PCC services existed in the United Kingdom at the start of the pandemic, with an increasing number of emergency clinics being opened rapidly throughout 2020. NHS England and local commissioners currently fund 90 specialist PCC clinics. Variability in service design and delivery across clinics still exists, with different staff numbers, specialist staff, funding levels, and the selection of PROMs changing over time as services develop and new research emerges. The C19-YRS PROM has been used widely across the UK since its development in Leeds and recommendation by NHS England in their national commissioning guidance for post-COVID services [19,20].

Specialist PCC clinics at Leeds Community Healthcare, Airedale, Northern Care Alliance (Salford Royal and Pennine Acute) and Liverpool University Hospitals NHS Trusts were the first 5 adopters of ELAROS’ DPROM platform. Each clinic had previously used the C19-YRS self-report questionnaire in one-to-one tele-assessments of patients in their PCC services prior to the digital platform’s launch in June 2021. Each clinic was using additional PROMs, such as the PHQ9, GAD7, Medical Research Council, Modified Fatigue Impact Scale, or the EQ-5D-5L, which were all made available on the DPROM platform to meet the request of each site to help take a deeper analysis of specific symptoms, quality of life, and cost-effectiveness analysis for patient care and in local service evaluation projects.

As more clinics have adopted the DPROM platform, additional PROMs have been added to the platform to support routine service, research projects, and to help validate new emerging PROMs used for the assessment of PCC and other long-term conditions.

### Designing Summary Reports

Given the complex, multivariate nature of PCC, patients are commonly advised to repeat their assessments at longitudinal time points to be defined by the clinic, with NHS England recommending that adult patients complete their outcome measures on first assessment, three monthly during follow-up or rehab support, and at discharge from the service [19].

Additional clinical measurements and demographics were requested to be collected through the platform to gain a better understanding of the patient’s overall health and to meet local, regional, and national reporting requirements to undertake routine service audits or evaluations of patients being seen in services and in research. Detailed metrics and summary data collected and processed by the DPROM platform are presented to staff in the clinical web portal to analyze internally to help guide conversations with people with PCC and deliver clinical care, with each tool displaying data in different ways.

It is a common requirement among health care organizations to maintain EHRs in a central system, therefore a mechanism was required to export data from the DPROM platform in an accessible and easy-read format to upload to local EHR. This clinical information can be compiled into a summary report and exported from the DPROM platform as a PDF which can be uploaded to the patient’s EHRs at any time, enabling staff to take a “snapshot” of the patient’s condition at different timepoints which can be stored permanently on local EHRs for other clinics to access. Alternatively, raw assessment data can be exported as a comma-separated values file for individual users or manually transcribed into the EHR platform, as different EHR systems accept different file formats.

### User-Centered Design of the Platform

The C19-YRS platform was adapted from ELAROS’ DBD platform which had previously undergone extensive user testing to refine the delivery mechanism and usability of the system to develop an effective, easy-to-use digital platform that served as the basis for a new app toward PCC. In 2020-2021, continence clinical specialists, patients, care home residents and staff, and members of the community at Aston University’s Research Centre for Healthy Ageing trialled the original DBD as part of a UK Research and Innovation funded Innovate UK project to evaluate the usability of the platform [23]. Feedback for the DBD app was positive, with 95% (n=19) of service evaluation participants stating the app was either “easy” or “very easy” to use, and 100% (N=20) stating they would be happy to use the app again.

This provided ELAROS with sufficient confidence to pivot their urology-focused system into PCC to develop an effective minimal viable product as a starting point for early PCC clinical adopters to rigorously test and help refine the platform for people with PCC and specialist clinics to develop version 1.0 of the DPROM platform during national lockdowns, achieving something that would normally take years to build from scratch in as few as 7 months.

ELAROS worked closely with people with PCC and multidisciplinary teams of clinicians, researchers, physios, therapists, and patient representatives on behalf of PCC patients at University of Leeds, Leeds Community Healthcare NHS Trust, Leeds Teaching Hospitals NHS Trusts, and Airedale NHS Foundation Trust to understand end user technical and usability requirements to develop a roadmap, implement the system into a test bed across various clinics, and iterate the design using continuous feedback from users.

The PCC clinic at Salford Royal NHS Foundation Trust (now the Northern Care Alliance NHS Trust) contacted ELAROS in January 2021 expressing an interest in the digital system being developed, and later joined the network to contribute invaluable support with end user testing with staff and patients, conducting a needs analysis, and supporting ELAROS to overcome regulatory challenges with information governance, clinical safety, and procurement.

Regular weekly or fortnightly Patient and Public Involvement or Patient Advisory Group groups had already been set up at a number of NHS sites since early in the pandemic to help inform clinics on how to develop local pathways and services to offer adequate care to patients, based on their collective understanding of a novel condition. These remote PPI groups at Leeds and Salford Royal NHS Trust served as the natural target to introduce the DPROM platform to gather early feedback and input to the discovery, design, and development phases of the DPROM platform to rapidly develop the platform ready for live service in June 2021.

## Results

### Results of the Requirement Analysis

The needs and requirement analysis after discussion with clinicians, researchers, and people with PCC suggested that the key requirements of a DPROM system include the following: (1) people with PCC should directly respond to the questionnaires on the system rather than having to depend on paper forms and having to send them back to the service or clinicians; (2) real-time assessment should be performed by the clinicians, rather than having to depend on the return of the forms from the patients, due to the administrative burden on the system of having to manage the returned forms; (3) feedback on the information collected from the questionnaires should be returned to the respondent in an easy-to-understand way, so that it helps them monitor and understand the course of the condition and is useful in self-management; (4) the system should include self-management resources or direct users to resources available elsewhere; (5) PROMs need to be PCC specific, succinct yet comprehensive, and not burdensome to the respondents; (6) PROM data should integrate with the existing EHRs in a standardized manner for every respondent; (7) consent from respondents should be gathered to use their data for approved research or service audits and evaluations; (8) user data should be automatically pseudonymized (ie, the removal of identifiable information) for use in research studies; and (9) an analysis of summary statistics should be generated for the entire cohort of users in the system in an easy-to-understand format for service providers and commissioners investing in the PCC service.

### Digital Platform Functionalities

The DPROM platform comprises 2 core components; an on the internet clinical web portal used by staff within the PCC clinic to oversee patients, analyze assessments, communicate with the patient, and extract data for permanent storage in EHRs; and a patient-facing app used to complete assessments, communicate with the clinic, and access rehabilitation resources between appointments.

**Figure 2:**
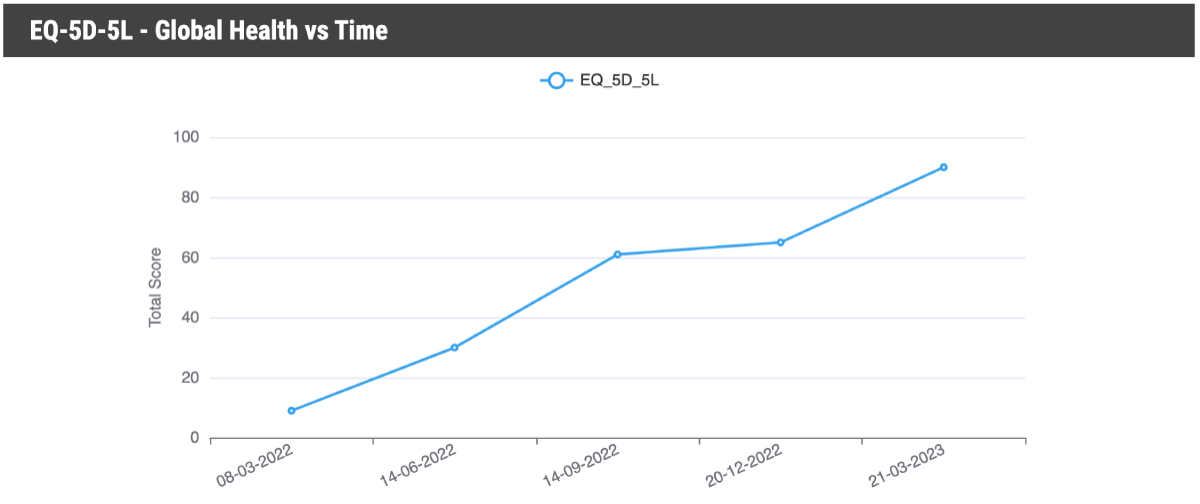
Example graph illustrating changes in depression scores using the PHQ-9. PHQ: Patient Health Questionnaire.

**Figure 3:**
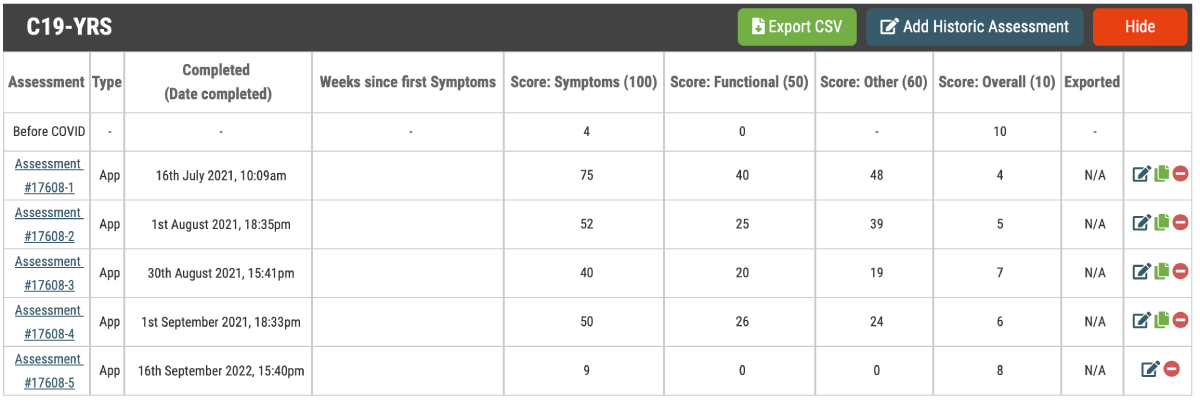
Example table illustrating change in C19-YRS scores over time. C19-YRS: Yorkshire Rehabilitation Scale.

The on the internet web portal hosted in the cloud, is the central digital “hub” for PCC clinics to access to register new patients who are referred to the clinic with an account, administer a selection of PROMs with preconfigured automatic reminders, analyze health data as it is received and processed into graphical (Figure 2) or tabular (Figure 3) format, communicate with the patient via 1- or 2-way messaging, and extract data ready for upload and permanent storage in the patient’s EHR managed by the clinic.

**Figure 4:**
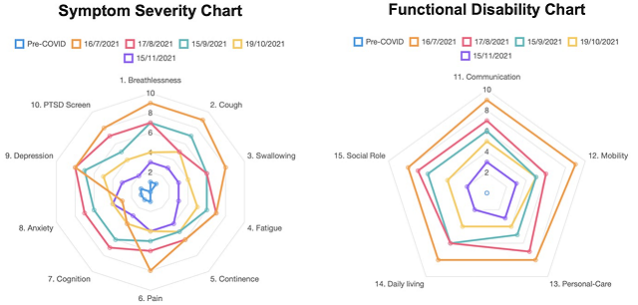
Digital patient-reported outcome measure platform radar plot display of severity of symptoms and functional disability. PTSD: posttraumatic stress disorder.

The platform generated a radar chart, or spider chart, to illustrate to the patient the multiple symptoms recorded in the C19-YRS and how they fluctuate over time (Figure 4). Radar charts are helpful in also enabling staff to draw comparisons between multiple items, identify outliers, and evaluate trajectories of symptom severity over time. The C19-YRS has 4 subscales concerned with the severity of patients’ key symptoms, functional limitations, overall health, and additional symptoms [13,18]. Questions 1-10 form the Symptom Severity Subscale and questions 11-15 form the Functional Disability Subscale, which are both presented as radar chart to the patient inside the smartphone app for self-monitoring and to evaluate progress over time, as well as to staff in the clinical web portal.

The patient-facing app can be accessed on mobile by the patient if they are confident enough with digital technology and their personal circumstances allow them access to a mobile device to self-report information about their symptoms and wish to access educational and rehabilitation resources available within the app for self-monitoring and self-management purposes. Alternatively, patients have an alternative option of accessing the same app on the internet via a web-based version, which helps patients who may struggle with reading difficulties on smaller phone screens, or those who do not have access to an adequate personal mobile device. Clinical staff, too, can access the web-based version to sign in on the patient’s behalf to complete assessments with a patient or their carer or guardian as part of a tele-assessment, which has proved useful to patients who may be digitally excluded from using a digital device independently, due to illness or socioeconomic reasons.

A research version of the clinical portal is also available to authorized staff to access pseudonymized data sets from patients who have given consent to sharing their data for appropriate means, for example as part of a regional service evaluation carried out by staff external to the clinic, or as part of national research projects such as the National Institute for Health and Care Research–funded LOCOMOTION project involving 11 clinical sites in 3 countries [24]. This automatic pseudonymization and provision of consent remotely through the platform is time-saving and less burdensome on research teams than traditionally using paper forms through the post and manually logging information into a computer.

**Figure 5:**
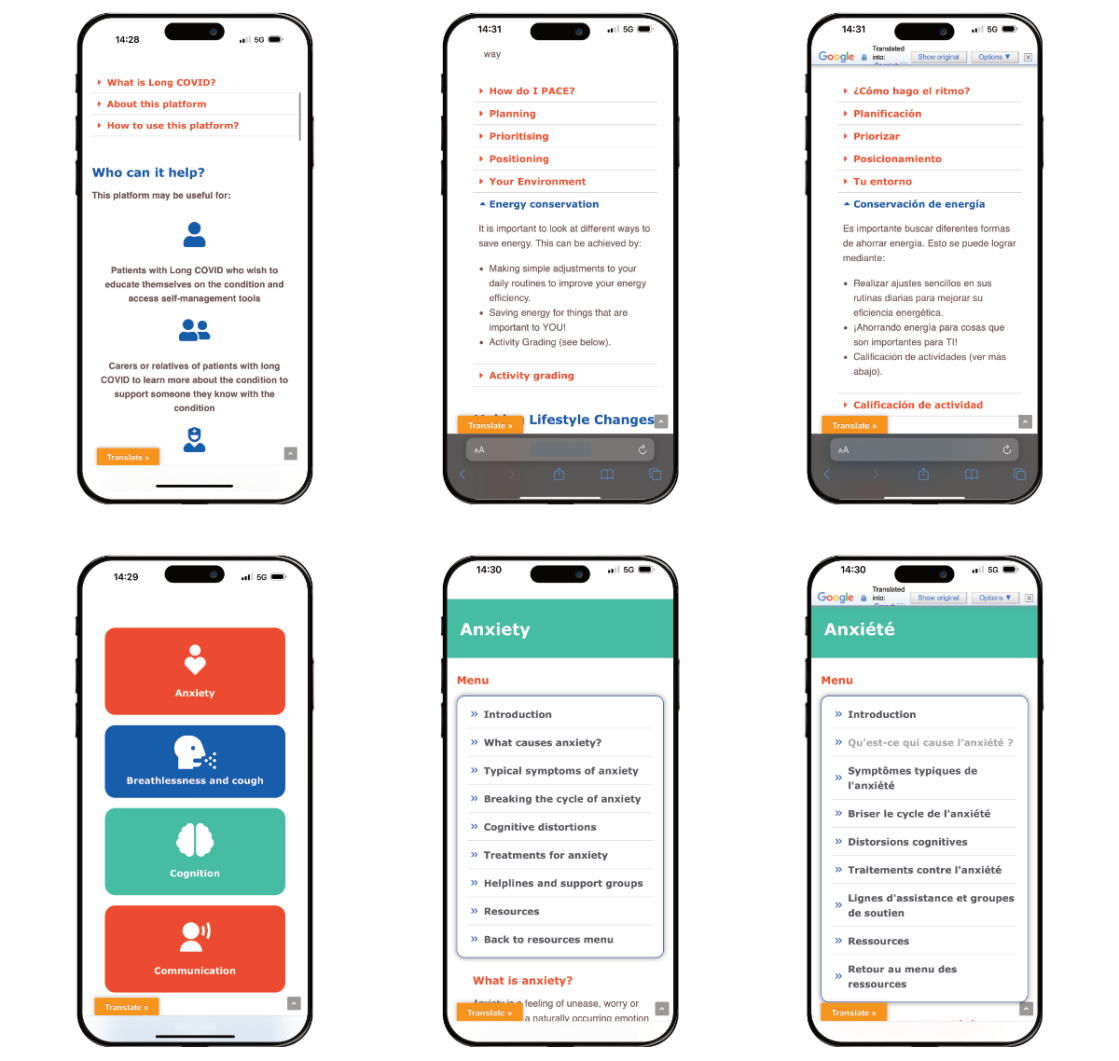
Digital patient-reported outcome measure platform support resources for self-management.

The patient app also incorporates numerous translatable support resources (Figure 5) to help patients and carers to educate themselves and access rehabilitation resources around different elements of their condition in an easy-read, mobile optimized or on the internet format. The resources curated and packaged by ELAROS have been contributed by various NHS trusts who developed these resources to support their patients. This provides assurance on content validity and clinical quality, promoting sharing of best practices and acquired knowledge on how clinics are approaching this new condition.

### PROMs Available on the Platform

The DPROM platform has more than 30 PROMs and related health questionnaires at the time of writing (Textbox 1), with additional measures being requested by clinics inside and outside the United Kingdom, some of which need further work with the scale developers to ensure licensing requirements of the scales are met.

**Digital patient-reported outcome measure platform questionnaires.**

- General Health Information Questionnaire
- Adapted Autonomic Profile (aAP)
- C19-YRS (Yorkshire Rehabilitation Scale)
- MC19-YRSm (Modified C19-YRS; modified Yorkshire Rehabilitation Scale)
- Brief Pain Inventory (BPI)
- Chalder Fatigue Scale
- Dyspnea-12 (D-12)
- EQ-5D-5L
- EQ-5D-Y
- Functional Assessment of Chronic Illness Therapy (FACIT) - Fatigue
- Generalized Anxiety Disorder (GAD-7)
- Health Economics Questionnaire - Baseline
- Health Economics Questionnaire - Follow Up
- Hope, Agency and Opportunity (HAO)
- Long COVID Friends and Family Test Survey
- Long-Term Conditions Questionnaire Short Form (LTCQ-8)
- Modified Fatigue Impact Scale (MFIS)
- Medical Research Council (MRC) Dyspnoea Scale
- Nijmegen Questionnaire
- Pain Catastrophizing Scale (PCS)
- Pain Detect Questionnaire
- Patient Health Questionnaire (PHQ-8; PHQ-9)
- Readiness to Return to Work
- Revised Childrens’ Anxiety and Depression Scale (RCADS - Child Reported)
- Revised Childrens’ Anxiety and Depression Scale (RCADS - Parent Reported)
- Short Revised Childrens’ Anxiety and Depression Scale (Child Reported)
- Short Revised Childrens’ Anxiety and Depression Scale (Parent Reported)
- Self-Efficacy Scale for Managing Chronic Pain
- Short Form Survey (SF-12; version 1)
- Short Form Survey (SF-36; version 1)
- Short Form Survey (SF-36; version 1 physical subscale)
- Symptoms Self-Efficacy Scale (SSEQ)
- Visual Analogue Pain Scale
- Vocational Rehabilitation Questionnaire
- Work and Social Adjustment Scale (WSAS)
- Widespread Pain Index-Symptom Severity (WPI-SS)

### Summary Report for Electronic Records

**Figure 6:**
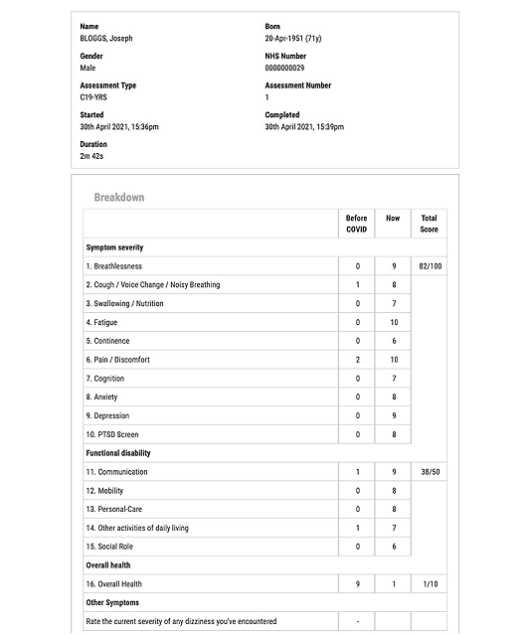
A sample C19-YRS summary report generated by the web portal. C19-YRS: Yorkshire Rehabilitation Scale; PTSD: posttraumatic stress disorder.

The platform generates summary reports that can be uploaded to patient records (Figure 6). Aggregate reports for the entire service caseload can also be generated which can be used for service evaluation of outcomes and sharing with national regulatory authorities such as NHS England.

### Usability Outcomes

The platform is currently used by 46 PCC centers in England, 2 centers in Scotland, and 1 center in Wales, servicing approximately 10,000 patients across 32 NHS trusts and health boards at the time of writing, with an additional 18 new centers in England, Scotland, and Australia working their way through governance which are due to come on board.

A sample of quotes (anonymized) from patients and staff using the system is summarized in Table 1.

**Table 1:** Sample of user quotes.

End user type	Digital patient-reported outcome measure component	Quote
Patient user	Smartphone app	“I found the app easy to download and very user friendly. I’m looking forward to using it more and intrigued as to the next steps, hoping it will help me to get the right support and care.”
Senior administrator (administrator user)	Overall platform	“The C19(YRS) app has literally been a game changer, reducing the patient’s waiting time massively.” “The app is user friendly and generally patients complete their C19-YRS on the day we send the app info out, resulting in their waiting time being reduced for their virtual appointment by at least 10-14 days.” “The patient information is so easy to take from the platform and add on to SystmOne and when we then require them to complete other measures it’s a super easy process to log back into the app and amend which requirements we need next from them. Postage and printing time and costs have been reduced massively, saving us several hours of work each week printing letters, posting out the paper questionnaires, asking patients to return it to us in an SAE and then having to scan the paper copy on to SystmOne.”
Community advanced practitioner (clinician user)	Patient-facing app	“From experience in the first wave, 88 patients were screened by phone by a member of the trust totalling on average of 1 hour each. With the number potentially now totalling a minimum of 10x this and with the need to complete 3x per patient the time taken and cost to the trust will be extensive.” “On average the app takes clinicians 10 minutes or less to complete an assessment with patients.”
Clinical service lead (clinician user)	Overall platform	“Lanarkshire have been so impressed with the capacity and capability the digital C19-YRS system brings to our Long Covid Rehabilitation Pathway. Since adoption, people with Long Covid have their initial screening three weeks faster, on average, meaning they are triaged and added to the waiting list 3 weeks sooner.” “Administration report a 90% reduction in time spent supporting screening. Clinicians have easy access to questionnaires and summary reports with a 50% reduction in the time taken to triage each person.” “Most importantly, our people with Long Covid on the pathway are spending less time and precious energy to complete the questionnaires. They have access to their own data and can track their own progress – this supports shared decision making regarding care planning, thus really helping the pathway optimise person-centred care. People with Long Covid also have easy access to evidence-based, self-management resources and information.” “The platform is intuitive to use for all concerned and there are multiple options for people who may have issues with digital access and literacy. There has been ample opportunity to tailor the platform to the needs of our service – changes have been quick and well-supported.” “On a population level, the data the system generates is crucial to build an understanding of the needs of people with Long Covid and evaluate whether our pathway is having an impact. This in turn informs the strategic direction of support for people with Long Covid in Lanarkshire, and across Scotland. The import and potential of a national dataset for Long Covid in Scotland cannot be overemphasised.”

## Discussion

This DPROM platform is the first PCC platform reported in the literature to record PCC symptom profile, condition severity, functional disability, and quality of life via the C19-YRS and other PROMs within the platform. Individual-level demographic medical information and details on the COVID-19 illness can be captured systematically. People with PCC complete the PROMs on their smartphones or web application for the information to be available on web portal for the HCPs to see and monitor the progress of the condition. The platform generates easy-to-understand scores, radar plots, and line graphs for people with PCC to self-monitor their condition and assess response to interventions. Clinics can configure a suite of PROMs based on their local and national service and commissioning requirements, and support research studies which require large-scale data collection using PROMs.

The initial feedback from users of DPROM platform (Table 1) suggests it has been received well by people with PCC and PCC service staff. The feedback suggests the administration process for managing a large number of patients has become streamlined and far more efficient than using paper forms for PROMs. DPROM platform generates summary reports for clinical records and enables automatic aggregate data analysis for services to undertake service evaluation and cost-effectiveness analysis. The ongoing research studies using the platform will be reporting on outcomes soon [24]. These studies will also provide more information on the psychometric properties of PROMs (such as severity type, responsiveness, and clinically significant change in scores) which can be incorporated into the summary reports.

Multiple studies have explored the use of digital patient reported outcomes in other conditions [25]. Some studies have reported nonuse rates to be as high as 72% [26,27]. The reported reasons for not engaging with technology are manifold: (1) health problems affecting their ability to participate [28,29], (2) emotional distress when reporting their symptoms and being reminded about their illness [30,31], (3) getting better and having no symptoms to report [32,33], (4) not being interested [34,35], (5) difficulty finding time in busy daily life [30,36,37], (6) not seeing any personal benefit by participating [30], (7) lack of clinical input and interaction with the clinician providing care [25], (8) questionnaires being burdensome, (9) technical problems with the system or platform [26,34], and (10) data security concerns and passive data collection [36,37].

The widespread use of a variety of PROMs in PCC can present challenges to (1) people with PCC, who may have “questionnaire burnout” alongside fatigue and brain fog from their condition; (2) clinics, most of which are already overrun and overstretched, making it difficult to manage, track, and assess multiple PROMs over time; and (3) service audit and research teams, who are likely to find it difficult to compare outcomes across multiple patient cohorts when there is variability in PROMs used. There is an urgent need to develop a core set of condition-specific PROMs used consistently in all clinics and a WHO working group is already undertaking this task [10,38]. The DPROM platform also needs to be adapted and tested in other long-term conditions [39].

The DPROM platform is likely to face challenges of use and compliance as experienced by other digital patient-reported outcomes interventions reported in the literature. As PCC is a novel condition with long-term outcomes not definitely known and interest from services and national regulatory authorities, there is likely to be better engagement from users. The use of the platform in multiple ongoing research studies is also likely to provide quality assurance to users to engage with the platform. In the near future, we will report findings of these studies, including the national NHS England service evaluation of PCC services, using the platform. These studies will inform the further development of the DPROM platform to be able to inform the best use of such technology in managing the novel condition.

## Abbreviations

DBD: Digital Bladder Diary
DPROM: digital patient-reported outcome measure
EHR: electronic health record
HCP: health care professional
NHS: National Health Service
PCC: post–COVID-19 condition
PREM: patient-reported experience measure
PROM: patient-reported outcome measure
WHO: World Health Organization
